# Comparative Analysis of LiMPO_4_ (M = Fe, Co, Cr, Mn, V) as Cathode Materials for Lithium-Ion Battery Applications—A First-Principle-Based Theoretical Approach

**DOI:** 10.3390/nano12193266

**Published:** 2022-09-20

**Authors:** Sayan Kanungo, Ankur Bhattacharjee, Naresh Bahadursha, Aritra Ghosh

**Affiliations:** 1 Department of Electrical and Electronics Engineering, Birla Institute of Technology and Science-Pilani, Hyderabad Campus, Hyderabad 500078, India; 2 Materials Center for Sustainable Energy & Environment, Birla Institute of Technology and Science-Pilani, Hyderabad Campus, Hyderabad 500078, India; 3 Faculty of Environment, Science and Economy (ESE), Renewable Energy, Electric and Electronic Engineering, University of Exeter, Penryn TR10 9FE, UK

**Keywords:** lithium-ion battery, cathode material, LiMPO_4_, olivine structure, lithium transport, first-principle calculations, density functional theory

## Abstract

The rapidly increasing demand for energy storage has been consistently driving the exploration of different materials for Li-ion batteries, where the olivine lithium-metal phosphates (LiMPO_4_) are considered one of the most potential candidates for cathode-electrode design. In this context, the work presents an extensive comparative theoretical study of the electrochemical and electrical properties of iron (Fe)-, cobalt (Co)-, manganese (Mn)-, chromium (Cr)-, and vanadium (V)-based LiMPO_4_ materials for cathode design in lithium (Li)-ion battery applications, using the density-functional-theory (DFT)-based first-principle-calculation approach. The work emphasized different material and performance aspects of the cathode design, including the cohesive energy of the material, Li-intercalation energy in olivine structure, and intrinsic diffusion coefficient across the Li channel, as well as equilibrium potential and open-circuit potential at different charge-states of Li-ion batteries. The results indicate the specification of the metal atom significantly influences the Li diffusion across the olivine structure and the overall energetics of different LiMPO_4_. In this context, a clear correlation between the structural and electrochemical properties has been demonstrated in different LiMPO_4_. The key findings offer significant theoretical and design-level insight for estimating the performance of studied LiMPO_4_-based Li-ion batteries while interfacing with different application areas.

## 1. Introduction

Over the last two decades, the increasing concerns about global warming, rapidly depleting international reserves of fossil fuels, and a growing presence of portable electronics in different application areas have primarily driven the ever-increasing requirement for clean and efficient energy-storage systems [1,2,3,4]. In this context, the lithium (Li)-ion battery demonstrates steadily growing commercial footprints as an efficient energy-storage element owing to its high energy density, low self-discharge property, high open-circuit voltage, and long lifespan [3,4]. Specifically, the high-energy density of Li-ion batteries holds considerable promise as the power source of hybrid or fully electric vehicles in the background of reducing CO_2_ and other greenhouse gas emissions and cutting down fossil-fuel consumption in the automotive industry [3,5]. However, like any other energy-storage device, the performance of Li-ion batteries also significantly depends on the material properties of their constituent elements, specifically electrodes.

Some of the significant challenges involving electrode design—and thereby lifespan degradation of high-energy-density Li-ion batteries—include damage, fracture, and diffusion-induced stress in the electrodes with the Li-ion intercalation/de-intercalation [6,7] chemo-mechanical degradation of the electrodes [8], the co-existence of electrochemical reaction, and irradiation in the extreme environment leading to compromise of operational integrity of the electrodes [9]. Therefore, a renewed research interest has been observed in enhancing the performance of Li-ion batteries by integrating novel or emerging materials and their hybrids in the cathode and anode design [2,3,10]. In this context, the density-functional-theory-based first-principle-calculation approach has emerged as a highly efficient computational technique for estimating the potential of different emerging/novel material systems for the Li-ion battery designs, which can efficiently guide as well as complement experimental exploration in this direction [4,6]. The material specifications of cathode materials significantly influence the overall performance of Li-ion batteries, leading to growing research interest in theoretically exploring different material systems as well as material engineering strategies for cathode-design optimization [4,11].

The olivine LiMPO_4_ material family from the Pnma space group presents some of the most promising cathode materials for Li-ion battery applications, which are also commercially adopted (such as LiFePO_4_ and LiCoPO_4_) for battery design [12]. In this context, considerable research has recently been observed to optimize the relevant material properties of different LiMPO_4_ materials, where the explorations based on the first-principle calculation remain at the forefront of such research. In one of the early works in this direction, the Li diffusion was theoretically analyzed in different LiMPO_4_ (M = Mn, Fe, Co, and Ni) structures, where the diffusivity is typically underestimated compared to experimental reports [13]. The incorporation of atomistic defects in the LiMPO_4_ (M = Mn, Fe, Co, and Ni) structure reproduces a more reliable assessment of Li diffusion and establishes a curved Li-diffusion pathway across the Li channel along the [10] crystallographic directions [14,15]. Specifically, the presence of Li/M antisite defects in the lattice significantly affects the Li diffusion in LiMPO_4_ (M = Fe) [14] and may lead to inter-channel hopping [16]. The bulk diffusion in Li is affected by the presence of intrinsic strain as well as Li concentration in LiMPO_4_ (M = Fe) lattices [17]. Furthermore, the formation of the interface in Li_1−x_MPO_4_ (M = Fe) plays an important role in the faster charging/discharging in Li-ion batteries [5]. In this effect, the bulk and surface Li diffusion in the Fe-doped LiMPO_4_ (M = Co) structure has been theoretically studied, which reveals that the (010) surface demonstrates the lowest surface energy, wherein the Li-ion diffusion barrier can be notably reduced by Fe doping [18]. The doping in olivine LiMPO_4_ structures has been identified as a highly efficient approach for engineering the material properties of different LiMPO_4_ materials. In this context, the theoretical investigation suggests that the introduction of Ni and Fe co-doping presents a promising route to optimizing the electrochemical properties of LiMPO_4_ (M = Mn) [19]. A similar exploration suggests that the Mn doping in LiMPO_4_ (M = Co) can significantly influence the electrochemical activities of the material [20]. Apart from doping, the formation of hybrid LiM_x_N_1−x_PO_4_ with different metals is another promising material engineering strategy for optimizing the electrochemical properties. In this context, a recent theoretical investigation reveals that LiFe_x_Mn_1−x_PO_4_ possesses considerable potential as a cathode for Li-ion battery design [21,22]. On the other hand, very recently, an extensive DFT-based investigation has been performed to investigate the different reaction routes, intrinsic defect formations, and influence of a wide variety of doping in LiMPO_4_ (M = Fe), offering a vital theoretical understanding of the synthesis process [23].

However, the majority of the theoretical investigations studied the electronic and electrochemical properties of one or few LiMPO_4_, emphasizing specific electrochemical aspects of these materials. To date, no systematical theoretical investigation has been observed on the different olivine phosphate-based cathode materials on their electrochemical and electrical properties. Consequently, in this work, a detailed first-principle-based theoretical investigation has been performed on the LiMPO_4_ (M = Fe, Co, Cr, Mn, and V), emphasizing the structural, electrochemical, and electrical properties. In this context, the comparative cohesive energy, M-O (L-O) bond length, M-O (L-O) charge transfer, diffusion activation barrier, hopping distance, diffusion coefficient, equilibrium potential, and open-circuit voltage as a function of state-of-charge for different cathode materials are emphasized in this work.

## 2. Materials and Computational Methodology

### 2.1. Battery Mechanism

In a conventional lithium-ion battery, the battery cell includes a positive electrode (cathode), a negative electrode (anode), and a non-aqueous liquid electrolyte. Conventionally, graphite is adopted for the anode material, whereas the non-aqueous liquid electrolyte constitutes of LiPF_6_ salt in suitable organic solvents [4]. The choice of cathode materials greatly influences the overall performance of the Li-ion battery, and, subsequently, a wide number of materials have been explored in this context [4,5,24,25]. In this work, different LiMPO_4_ materials are considered for Li-ion batteries while keeping the specifications of other constituents of the battery cell unchanged.

**Figure 1:**
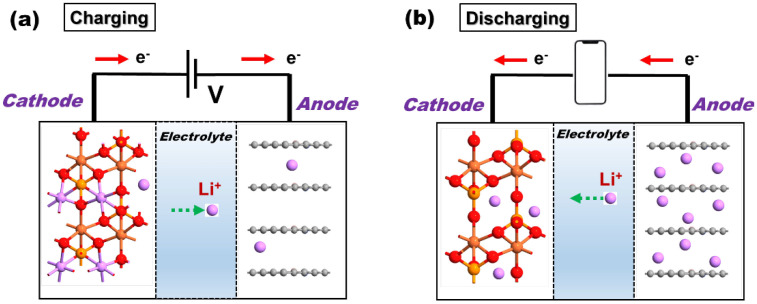
Schematic representation of Li-ion battery (**a**) charging and (**b**) discharging mechanisms.

During the charging cycle of the battery, a potential is applied across the electrodes leading to Li-ion extraction from the LiMPO_4_ cathode and subsequent ionic diffusion through electrolytes towards the anode. After reaching the anodes, the Li ions are intercalated between the layers of graphite, as shown in Figure 1a. Next, the Li ions return to the cathode from the anode during the discharging cycle. At the same time, electrons pass from the anode to the cathode through an external circuit acting as a power source for externally attached devices with the battery, as depicted in Figure 1b. The overall efficiency of the battery depends on the discharging cycle, whence the electrode reactions can be given as [4]:

At anode:

C_6_Li → 6C (Graphite) + x·Li^+^ + x·e^−^ (1)

At cathode:

Li_1−x_MPO_4_ + x·Li^+^ + x·e^−^ → LiMPO_4_ (2)

### 2.2. Material Specifications

**Figure 2:**
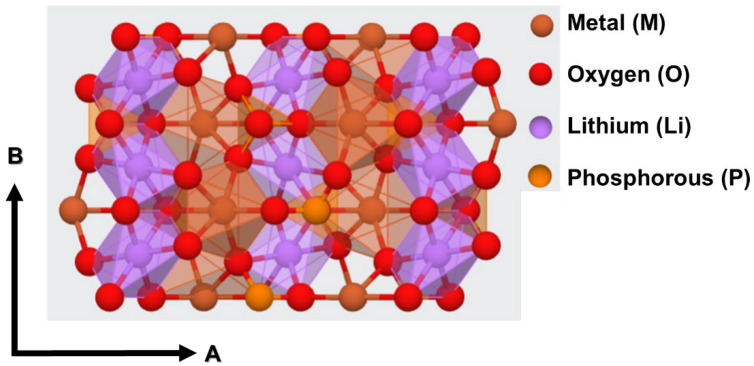
Schematic representation of the generic structure of LiMPO_4_.

The LiMPO_4_ family of phosphate materials demonstrates olivine structures and belongs to the orthorhombic Pnma space group [16,25]. In these three-dimensional structures, lithium (Li) is bonded with six oxygen (O) atoms to form LiO_6_ octahedra, which share corners with four equivalent MO_6_ octahedra and four equivalent PO_4_ tetrahedra, and, at the same time, share edges with two equivalent LiO_6_ octahedra and two equivalent MO_6_ octahedra, two equivalent PO_4_ tetrahedra, as depicted in Figure 2. A closer inspection of the crystal structure of LiMPO_4_ indicates that the LiO_6_ octahedra are corner-shared with each other and bridged by PO_4_ tetrahedra in parallel to the B-crystallographic axis, which creates a Li channel along the B-axis of LiMPO_4_. Furthermore, the individual channels are separated along the A-crystallographic axis by the MO_6_ octahedra, sharing both the edge and corner with LiO_6_ octahedra. Hence, there are two possible Li diffusion pathways through the B- and A-crystallographic axes. However, a number of previous theoretical reports conclusively established that the Li diffusions across individual channels along the B-crystallographic axis of LiMPO_4_ materials are more favorable compared to inter-channel hopping-dominated Li diffusion from one channel to another along the C-crystallographic axis [4,5,9,14,16,24,26].

### 2.3. Simulation Framework

In this work, the first principle calculations are performed using the commercially available Atomixtix Tool Kit (ATK) and Virtual Nano Lab (VNL) simulation packages from Synopsys Quantum Wise [27]. For the first-principle calculations, the linear combination of atomic orbitals (LCAO) PseudoDojo basis sets with the 3 × 5 × 6 Monkhorst-Pack grid for sampling the k points in the Brillouin zone and a density-mesh cutoff energy of 125 Hartree is considered [27]. The LCAO calculator of ATK provides a description of electronic structure using density functional theory (DFT) and the norm-conserving pseudopotentials, where the single-particle wave functions are expanded on the basis of numerical atomic orbitals [27]. It should be noted that the default energy-mesh cutoff significantly varies from one LiMPO_4_/MPO_4_ material system to another. Consequently, a uniform energy-mesh cutoff is considered in this work. The energy-mesh cutoff is taken as 125 Hartree, which is 1.25 times the highest default energy-mesh cutoff value (~100 Hartree) to ensure a proper trade-off between the reliability of the calculation and the overall computational cost. Moreover, the energy-mesh cutoff in the range of 100–200 Hartree in the LCAO basis set of ATK has also been reported previously in the literature [18,28,29,30].

**Figure 3:**
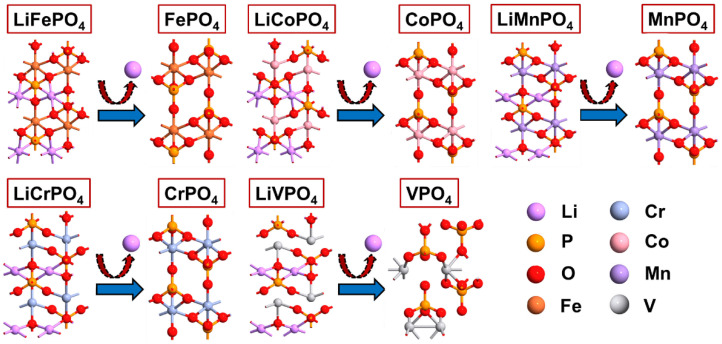
Schematic representation of optimized unit cells of different LiMPO_4_ (M = Fe, Co, Cr, Mn, and V) and MPO_4_ (M = Fe, Co, Cr, Mn, and V).

The unit cells of bulk LiMPO_4_ (M = Fe, Co, Cr, Mn, and V) and MPO_4_ (M = Fe, Co, Cr, Mn, and V) are first geometry optimized using the Generalized Gradient Approximation (GGA) method with the Perdew–Burke–Ernzerhof (PBE) Exchange-Correlation Functional and the Limited-Memory Broyden–Fletcher–Goldfarb–Shanno (LBFGS) Algorithm with a force and pressure tolerance of 0.001 eV/Å and 0.0001 eV/Å^3^, respectively [27]. The relaxed unit cells of different LiMPO_4_ (M = Fe, Co, Cr, Mn, and V) and MPO_4_ (M = Fe, Co, Cr, Mn, and V) are demonstrated in Figure 3.

The relaxed unit cells of LiMPO_4_ are modified to design initial and final material configurations for Li diffusion across a preferred crystalline axis and are subjected to further geometry optimization following the aforementioned procedure. The initial and final configurations are utilized for calculating Li diffusion across any preferred crystalline axis using the Ab-Initio Molecular Dynamics (AIMD)-based Nudge Elastic Band (NEB) simulation with the climbing image method by considering a 0.5 Å interval across the diffusion path between intermediate images [27]. It should be noted that in this work, the Li diffusion is studied across the Li channel (parallel to the B-crystallographic axis) of different LiMPO_4_ (M = Fe, Co, Cr, Mn, and V) materials, which is considered as the predominant Li transport direction. In this context, a number of existing reports argued that the GGA-PBE method could not accurately account for the electron-correlation effect, and the Hubbard GGA-PBE + U method improves the accuracy of the Li diffusion calculation for LiMPO_4_ materials [4,16,24]. The GGA + U method involves an empirical Hubbard U correction term for the individual material systems, where the results of the simulation are significantly influenced by the choice of U. However, choosing a set of appropriate empirical correction terms for each LiMPO_4_ material poses a significant challenge for the reliability of comparative performance estimations. Consequently, in this work, the GGA-PBE method is considered for both ground-state energy and AIMD-NEB calculations to ensure a uniform simulation platform for reliable qualitative performance estimations of different materials.

### 2.4. Parameter Definitions

The structural stability of the cathode materials in completely lithiated and completely de-lithiated phases are assessed from the cohesive energies (E_cohesive_lithiated_ and E_cohesive_de-lithiated_), which are defined as [31,32]

E_cohesive_lithiated_ = [E_LiMPO4_ − (4 × E_Lithium_ + 4 × E_Phosphorus_ + 16 × E_Oxygen_ + 4 × E_Metal_)]/28 (3)

E_cohesive_de-lithiated_ = [E_MPO4_ − (4 × E_Phosphorus_ + 16 × E_Oxigen_ + 4 × E_Metal_)]/24 (4)

where E_LiMPO4_ and E_MPO4_ are the ground-state energies of the LiMPO_4_ and MPO_4_ lattices. Furthermore, the E_Lithium_, E_Phosphorus_, E_Oxygen_, and E_Metal_ are the ground-state energies of isolated lithium, phosphorus, oxygen, and metal (iron, cobalt, chromium, manganese, and vanadium) atoms, respectively. The higher cohesive energies imply higher structural stability of the LiMPO_4_ and MPO_4_ lattices. The Li intercalation energy (E_Intercallation_) in Li_x_MPO_4_ is another essential performance matrix for cathode materials in Li-ion battery applications, which is defined between completely lithiated and single Li-ion de-lithiated phases of LiMPO_4_ lattices as follows [4]:

E_Intercallation_ = [E_Li4MPO4_ − E_Li3MPO4_ − E_Lithium_] (5)

where E_Li4MPO4_ and E_Li3MPO4_ are the ground-state energies of the completely lithiated and single Li-ion de-lithiated (averaged over Li position) LiMPO_4_ lattices.

The Li diffusion within olivine LiMPO_4_ structures is assessed in terms of intrinsic diffusion coefficient (D), which can be calculated from the atomic scale behavior using the framework of transition state theory under one-dimensional transport as follows [5,9]:

D = a^2^ × ν* × exp(−E_act_/K_B_T) (6)

where a, ν*, E_act_, K_B_, and T are the hopping distance, attempt frequency, diffusion activation energy, Boltzmann constant, and absolute temperature, respectively. In this context, it should be noted that the diffusion activation energy represents the potential barrier that has to be surmounted by the Li-ion during diffusion in LiMPO_4_. Furthermore, several definitions of attempt frequency in the context of diffusion exist in the literature [33,34]. However, the attempt frequency can be simply obtained by considering every vibration of a diffusing atom as an attempt, which allows defining the attempt frequency as the averaged vibrational frequency [33]. In this work, the diffusion coefficient is calculated at room temperature (T = 300 K), and a standard and uniform attempt frequency of ν* = 10^12^ Hz is considered from literature in the context of LiMPO_4_ materials [13]. Furthermore, both the hopping distance and activation barriers are calculated from the NEB simulated Li diffusion between the neighboring atomic sites across the specified crystallographic axis.

The equilibrium cell potential (V_eq_) of a Li battery with a specific LiMPO_4_ cathode material can be reasonably estimated from the first-principle calculation by ignoring the contributions of vibrational and configurational entropy to the cell potential at room temperature as follows [4]:

V_eq_ = [−(E_LiMPO4_ − E_MPO4_ − 4 × E_Lithium_)]/4q (7)

where q is the electronic charge. The equilibrium cell potential in this work is calculated considering the completely lithiated and completely de-lithiated phases of cathode materials [4].

Finally, the open-circuit voltage (V_OCV_) of the Li-ion battery cell is calculated for different state-of-charge (SOC) levels using the Nernst Potential Equation as follows:

V_OCV_ = V_eq_ + (2 × R × T/F) × ln[SOC/(1 − SOC)] (8)

where R is the molar gas constant, and F is Faraday’s number.

Equation (8) offers a quantitative estimation of the electrical characteristics of the battery, wherein the V_OCV_ can be determined under the variation of SOC. The V_OCV_-SOC characteristics indicate the available potential of a battery at different charging states (SOC). In this work, the range of SOC has been considered from 10% to 90% limit, which is a standard for Li-ion batteries for ensuring safe operation, i.e., avoiding the over-charge and over-discharge conditions.

## 3. Results and Discussion

This work discusses the comparative structural, electrochemical, and electrical properties of different LiMPO_4_ materials in the following subsections.

### 3.1. Structural Properties of LiMPO_4_ (M = Fe, Co, Cr, Mn, and V)

**Figure 4:**
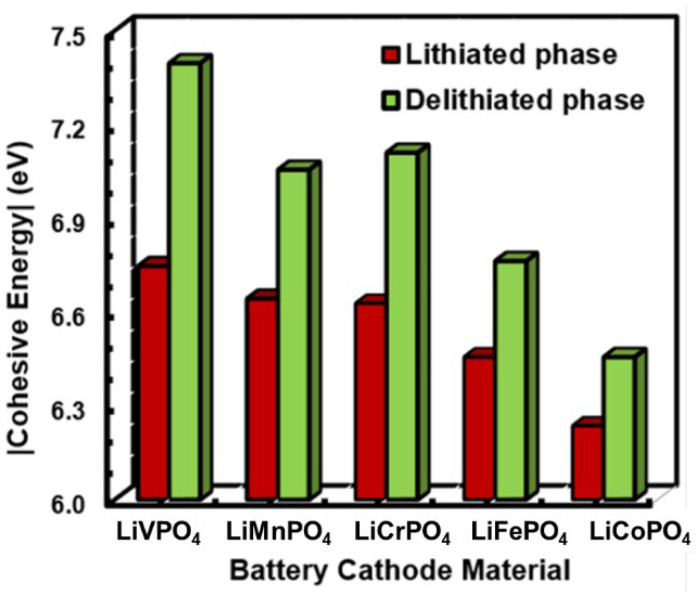
Comparative plots of cohesive energies in lithiated and de-lithiated phases for different LiMPO_4_ cathode materials.

First, the relative structural stability of individual LiMPO_4_ and MPO_4_ materials are analyzed from the cohesive energies and illustrated in Figure 4. The results indicate that, generally, the cohesive energy is considerably higher in the delithiated phase compared to the lithaited phase. Furthermore, the highest and lowest structural stability can be found for vanadium- and iron-based olivine phosphates, both in their lithiated and delithiated phases. However, the smaller difference in the cohesive energies between the lithiated and delithiated phases indicates the suitability of Li insertion in the olivine phosphate matrices [35]. Consequently, the LiCoPO_4_ and LiFePO_4_ suggest a superior cathode performance, whereas the least favorable performance can be expected from LiVPO_4_.

Next, the structural properties of LiMPO_4_ materials are estimated in terms of lattice vectors, lithium (Li)-oxygen (O) bond length, and Li atomic charge, which is tabulated in Table 1 and Table 2.

Table 1 indicates that the largest unit-cell lattice vector can be found for LiVPO_4_. In contrast, the smallest and the most comparable lattice vectors can be found in LiFePO_4_ and LiCoPO_4_. This trend can be attributed to the fact that the largest atomic radius of Vanadium (1.71 Å [36]) spatially expands the VO_6_ octahedra and thereby increases the volume of the entire unit cell of LiVPO_4_. In contrast, the smaller atomic radii of cobalt (1.52 Å [36]) and iron (1.56 Å [36]) restrict the volume of CoO_6_ and FeO_6_ octahedra, which leads to the smaller unit cell lattice vectors in LiFePO_4_/LiCoPO_4_. On the other hand, intermediate atomic radii of manganese (1.61 Å [36]) and chromium (1.66 Å [36]) result in intermediate lattice vector values in LiCrPO_4_ and LiMnPO_4_ compared to LiVPO_4_ and LiFePO_4_/LiCoPO_4_, as depicted in Table 1. Such distinctly different lattice vectors in LiMPO_4_ are expected to significantly influence the octahedral structure of LiO_6_ and thereby the Li-O bond strengths in different LiMPO_4_ materials, which are illustrated in Table 2. The larger values of unit cell lattice vector expand the LiO_6_ octahedra, and a relatively larger bond length (2.21 Å) can be found in LiVPO_4_. In contrast, the smaller unit cell lattice vector leads to relatively smaller bond lengths (2.13 Å) in LiFePO_4_ and LiCoPO_4_. Interestingly, the Mulliken charge accumulation on the Li atom strongly depends on the lattice vector and Li-O bond length in LiO_6_ octahedra, where an expanded bond length increases the Mulliken charge on Li, suggesting a weaker covalent bond formation. In this context, LiVPO_4_ exhibits the weakest Li-O bond strength, whereas the strongest bond strength can be found in LiFePO_4_ and LiCoPO_4_. In essence, the structural analysis offers crucial insight into the Li-O bond strength of LiO_6_ octahedra in different olivine LiMPO_4_ materials, which can be exploited for analyzing the comparative electrochemical properties of LiMPO_4_. It should be noted that the experimentally observed lattice constants of LiFePO_4_ (a = 10.35 Å, b = 6.02 Å, c = 4.70 Å) [37], LiCoPO_4_ (a = 10.20 Å, b = 5.92 Å, c = 4.70 Å) [38], and LiMnPO_4_ (a = 10.44 Å, b = 6.09 Å, c = 4.75 Å) [39] are in reasonable agreement with theoretically calculated values in this work.

### 3.2. Electrochemical Properties of LiMPO_4_ (M = Fe, Co, Cr, Mn, and V)

The electrochemical properties of LiMPO_4_-based cathode materials are appreciated from the Li intercalation energies in the lattice, as well as the Li diffusion process along the most favorable diffusion path, which is across the Li channels parallel to the B-crystallographic axis.

**Figure 5:**
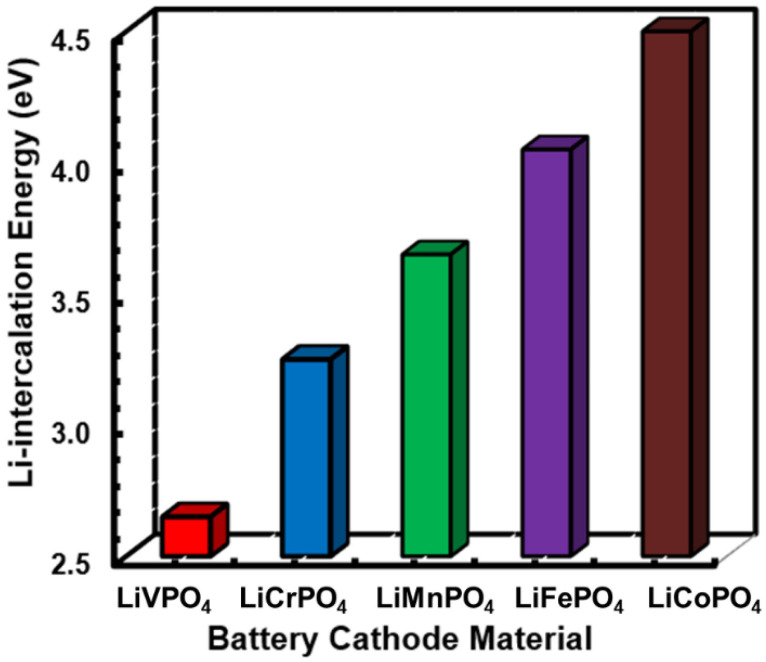
Comparative plots of lithium intercalation energies defined between completely lithiated and single Li-ion de-lithiated phases of the LiMPO_4_ lattice.

First, the relative Li intercalation energies for different cathode materials are depicted in Figure 5. Subsequently, a notably small (<3 eV) and large (>4 eV) Li intercalation energy can be found in LiVPO_4_ and LiFePO_4_/LiCoPO_4_, respectively. This trend can satisfactorily be co-related with the Li-O bond strength analysis that was performed in the previous subsection. Specifically, the relatively stronger covalent bonding between Li and O in LiFePO_4_ and LiCoPO_4_ makes it less favorable to remove one Li ion from these lattices, resulting in comparatively larger intercalation energies, as discussed previously. In contrast, the relatively weaker covalent Li-O bonding ensures a smaller Li intercalation energy in LiVPO_4_. In this line, the intermediate Li-O bond strength results in intermediate intercalation energies in LiMnPO_4_ and LiCrPO_4_. It should be noted that the calculated Li intercalation energy of 4.05 eV in LiFePO_4_ is slightly overestimated compared to the experimentally observed 3.50 eV value [5]. However, the theoretical prediction can be further improved by adopting more reliable DFT methods like the GGA-PBE + U [4].

**Figure 6:**
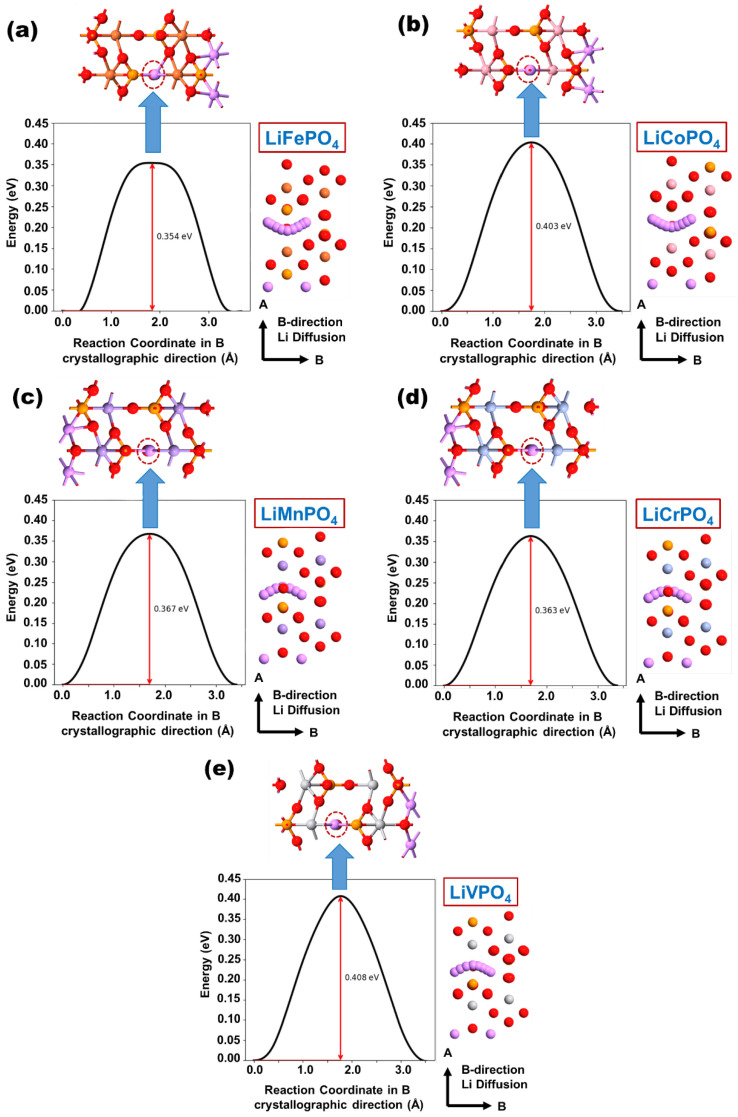
Plots of Li diffusion activation barrier along B-crystallographic axis and corresponding overlay image of Li diffusion for (**a**) LiFePO_4_, (**b**) LiCoPO_4_, (**c**) LiMnPO_4_, (**d**) LiCrPO_4_, and (**e**) LiVPO_4_.

Next, the Li diffusion energy profiles and corresponding Li migration pathways between two neighboring Li sites in different LiMPO_4_ are analyzed and are depicted in Figure 6. Figure 6 indicates that, in general, the Li diffusion between two neighboring Li-sites followed a curved migration pathway across the Li channel (as depicted in Figure 2). The results predict Li diffusion is essentially one-dimensional with a zigzag pathway across the AB-crystallographic plane of the lattices. It should be noted that the shapes of Li diffusion trajectories and the Li diffusion barriers in different LiMPO_4_ is consistent with the literature [4,5,9,14,16,24,26]. From the result, it is apparent that the atomic and chemical environment of the Li diffusion path significantly influences the Li diffusion activation barrier and hopping distance between two neighboring Li atom positions. Consequently, the structural configurations of different LiMPO_4_, which represent the maximum Li diffusion activation barrier have also been considered in Figure 6. In the structural configuration leading to the maximum Li diffusion activation barrier height, the Li forms three bonds with the one neighboring metal atom and two neighboring oxygen atoms, which is distinctly different from any other structural configurations in Li diffusion path. Interestingly, it has been found that the Li-O bond length remains comparable, i.e., in the range of 1.85 Å–1.87 Å and 1.89 Å–1.90 Å, irrespective of the particular olivine structure. On the other hand, the Li-M bond length more noticeably varies within the range of 2.53 Å–2.63 Å for different LiMPO_4_. This suggests that the M atom significantly influences the Li diffusion process in each LiMPO_4_ material. Correspondingly, a distinctly different Li diffusion activation barrier height (across the B-crystallographic axis) and energy-distance curvature of the diffusion barrier can be found in each olivine structure.

The calculated Li activation energy of 0.35 eV in LiFePO_4_ is in reasonable agreement with the previously GGA-PBE-based theoretically reported values in the range of 0.27–0.29 eV [5,9]. In contrast, the theoretically calculated value is notably underestimated compared to the experimentally observed range of 0.55–0.65 eV [40]. In this context, the adoption of the GGA-PBE + U method can further improve the theoretical estimation of activation energy compared to the GGA-PBE. However, it is worth mentioning that compared to experimental reports, a similar underestimation of activation energy has also been observed in previous theoretical reports, which is often correlated with the presence of non-idealities and their subsequent influence on Li-atom diffusion in the LiMPO_4_ structures [4].

**Figure 7:**
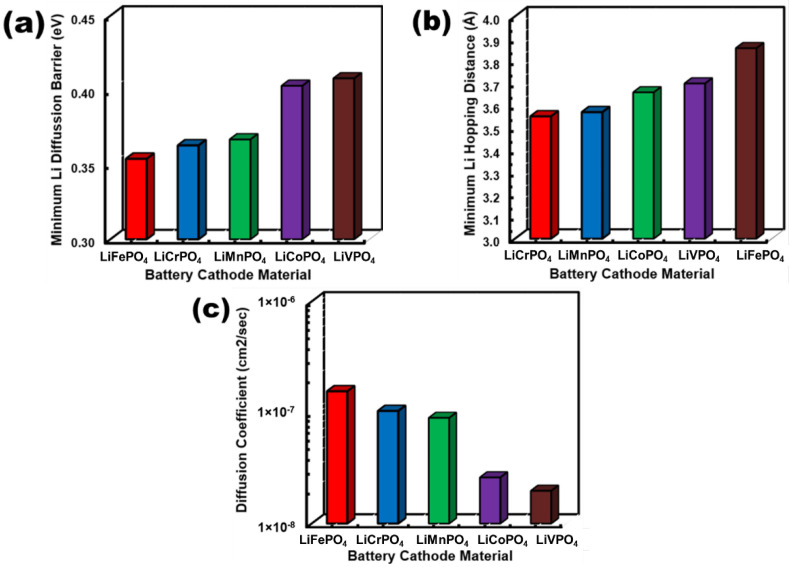
Comparative plots of (**a**) minimum activation barrier, (**b**) minimum hopping distance, and (**c**) intrinsic diffusion co-efficient for Li diffusion across Li channel (parallel to B-crystallographic axis) for different LiMPO_4_ cathode materials.

Next, the Li atom diffusion across the B-crystallographic axis is quantified in terms of diffusion activation barrier height, hopping distance, and intrinsic diffusion coefficient, and are depicted in Figure 7a–c, respectively. The LiFePO_4_ exhibits the lowest diffusion activation barrier height, which is slightly lesser than the barrier height of LiCrPO_4_ and LiMnPO_4_. Moreover, notably larger diffusion activation barrier height can be found in LiCoPO_4_ and LiVPO_4_. In contrast, the hopping distance indicates a different trend, where the smallest and largest hopping distance B-crystallographic axes can be found in LiCrPO_4_ and LiFePO_4_, respectively. However, the intrinsic diffusion coefficient (within the range of 0.354 eV–0.408 eV) exhibits an exponential dependence over activation barrier height, as depicted in Equation (6). Moreover, a relatively smaller range of variation in the hopping distances (3.55 Å–3.86 Å) can be found in different LiMPO_4_. Consequently, the activation barrier height effectively dominates over the diffusion coefficients. Subsequently, the smallest and largest intrinsic diffusion coefficient can be found in LiVPO_4_ and LiFePO_4_, where nearly an order of magnitude difference (1.97 × 10^−8^ cm^2^/s–1.55 × 10^−7^ cm^2^/s) can be found between these two olivine structures. Moreover, the LiCrPO_4_ and LiMnPO_4_ also demonstrate slightly smaller diffusion coefficients (1.03 × 10^−8^ cm^2^/s and 8.94 × 10^−8^ cm^2^/s) compared to LiFePO_4_, whereas LiCoPO_4_ exhibits a diffusion coefficient (2.60 × 10^−8^ cm^2^/s) that is comparable to LiVPO_4_.

### 3.3. Electrical Characteristics of LiMPO_4_ (M = Fe, Co, Cr, Mn, and V)

**Figure 8:**
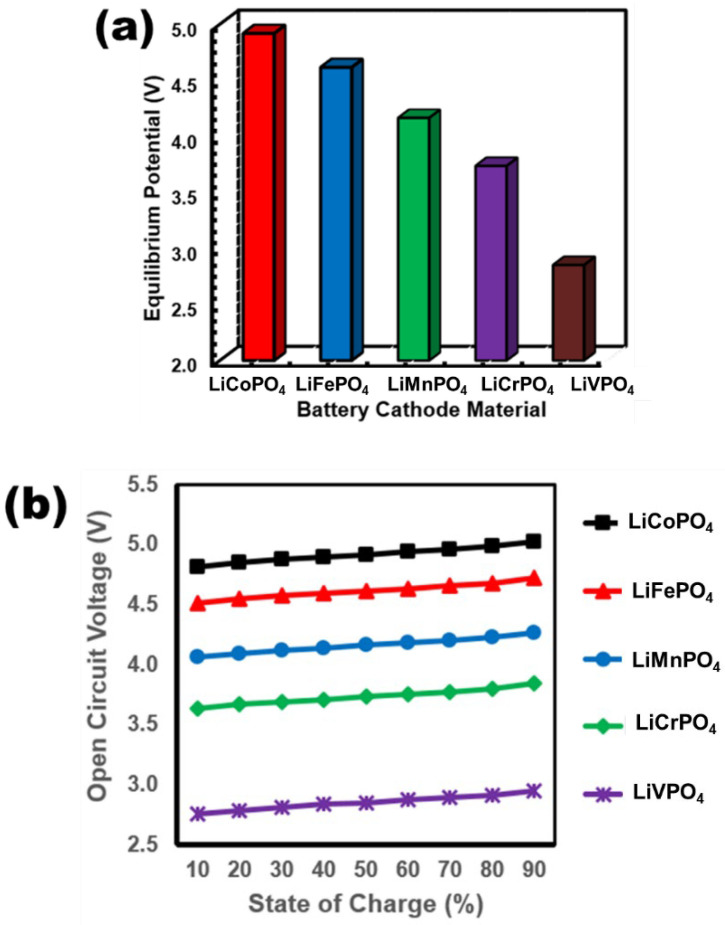
Comparative plots of (**a**) equilibrium potential and (**b**) open-circuit voltage as a function of state of charge for different LiMPO_4_ cathode materials.

In this work, five different and compatible metal (M = Fe, Co, Cr, Mn, and V) based samples of Li-ion battery cells have been simulated to analyze their electrical characteristics. It is important to analyze the battery’s electrical characteristics [41] for further estimation of their performance while interfacing with different useful applications. As mentioned in Equation (7), the Nernst potential equation provides the battery open-circuit voltage (V_OCV_) as a function of the equilibrium potential (V_eq_). The V_eq_ significantly varies with the cathode material. Therefore, in this paper, to examine the electrical characteristics of 5 different olivine phosphate material-based cathodes, the V_eq_ has been presented in Figure 8a. Further, based on the principle of the Nernst equation, the V_OCV_ profile over the range of SOC (10–90%) has been determined for different LiMPO_4_ cathode materials, as shown in Figure 8b. It is to be noted from the simulation results that the V_eq_ and V_OCV_ of LiCoPO_4_ cell is the highest (V_OCV_ around 4.9 V/cell), and that of LiVPO_4_ attains the lowest value (V_OCV_ around 2.7 V/cell). In the presence of Vanadium, the Li diffusion barrier increases, resulting in the battery voltage attaining a lesser value. Interestingly, it is found in Figure 8 that for the LiFePO_4_ sample, the V_OCV_ attains a value of 4.6 V/cell, which is close to the maximum value possessed by LiCoPO_4_. Also, the diffusion barrier for LiFePO_4_ is lesser compared to that of the other LiMPO_4_ battery samples. Therefore, due to these properties of LiFePO_4_, it has been reasonably popular among the other LiMPO_4_ batteries in electrical power system applications where high energy density and fast charge-discharge capacity are concerned. It should be noted that the experimentally demonstrated cell voltage of LiFePO_4_ typically lies between 3.5 V (10% SOC) to 4.2 V (90% SOC) [42]. However, the theoretically calculated V_OCV_ exhibits a range of 4.51 V (10% SOC) to 4.72 V (90% SOC). This overestimation of V_OCV_ in this work can be attributed to both the choice of the GGA-PBE DFT method instead of GGA-PBE + U as well as inherent theoretical limitations in accounting for practical factors like self-discharge drop.

## 4. Conclusions

The work presents an extensive first principle calculation based on theoretical investigations of the comparative electrochemical and electrical properties of different olivine lithium-metal phosphates for battery-storage applications. The results indicate that the presence of iron (Fe), cobalt (Co), manganese (Mn), chromium (Cr), and vanadium (V) atoms in the olivine phosphate structure distinctly influence structural stabilities and Li-atom diffusion across the Li channel, as well as battery open-circuit potential. It has been found that the lattice vector and, thereby, the Li-O bond specifications in LiO_6_ octahedral primarily determine the Li intercalation energies in different LiMPO_4_ materials. Furthermore, the chemical-bond formation of Li with specific metal atoms in the lattice during Li-atom diffusion effectively determines the lithium activation barrier height and curvature. Typically, LiVPO_4_ demonstrates a notably smaller Li intercalation energy of 2.65 eV compared to LiFePO_4_ and LiCoPO_4_, which exhibits intercalation energies in the range of 4.00–4.50 eV. Specifically, despite its lowest intercalation energy, the potential of LiVPO_4_ is compromised by its notably smaller Li intrinsic diffusion coefficient as well as low equilibrium voltage. In contrast, the notably higher Li intrinsic diffusion coefficient of 1.55 × 10^−7^ cm^2^/s and equilibrium voltage of 4.62 V can be achieved in LiFePO_4_, in contrast to the Li intrinsic diffusion coefficient in the range of 1.97 × 10^−8^–2.60 × 10^−8^ cm^2^/s in LiVPO_4_ and LiCoPO_4_ and equilibrium voltage of 2.85 V in LiVPO_4_. However, the relatively higher intercalation energy remains a limiting factor in LiFePO_4_. On the other hand, except for the higher equilibrium voltage of 4.92 V in LiCoPO_4_, both the higher intercalation energy and lower Li atom diffusivity present a severe bottleneck for this material for cathode design. However, both LiCrPO_4_ and LiMnPO_4_ represent a reasonable trade-off between intercalation energy (in the range of 3.25–3.65 eV), intrinsic diffusion coefficient (in the range of 1.03 × 10^−7^–8.94 × 10^−8^ cm^2^/s), and equilibrium voltage (in the range of 3.74–4.16 V). The trend suggests that none of the studied LiMPO_4_ is simultaneously co-optimizing both intercalation energy and diffusion coefficient to achieve an unequivocally faster charging/discharging in the cathode. Subsequently, a dedicated material engineering approach is necessary to optimize the overall charge-discharge property of LiMPO_4_ materials for Li-ion battery storage.

## Figures and Tables

**Table 1:** The Lattice Constants of Different Olivine LiMPO_4_.

Materials	Lattice Constant, a (Å)	Lattice Constant, b (Å)	Lattice Constant, c (Å)
LiFePO_4_	9.851	5.773	4.672
LiCoPO_4_	9.885	5.882	4.702
LiMnPO_4_	9.953	5.830	4.689
LiCrPO_4_	10.041	5.882	4.707
LiVPO_4_	10.276	5.981	4.709

**Table 2:** The Li-O bond length and Mulliken Charge on Li in Olivine LiMPO_4_.

Materials	Li-O Bond Length Range (Å)	Mulliken Charge on Li (e^−^)
LiFePO_4_	2.07–2.13	0.009
LiCoPO_4_	2.09–2.13	0.009
LiMnPO_4_	2.07–2.17	0.052
LiCrPO_4_	2.07–2.20	0.054
LiVPO_4_	2.09–2.21	0.083

## Data Availability

The raw/processed data required to reproduce the above findings cannot be shared at this time, as the data also forms part of an ongoing study.
